# 71 Estimation of incidence of MIS-C in Cape Town, South Africa

**DOI:** 10.1093/rheumatology/keac496.067

**Published:** 2022-10-07

**Authors:** Claire Butters, Deepthi Abrahams, Raphaella Stander, Timothy Spraklen, Heidi Facey-Thomas, Liesl Zühlke, Helena Rabie, Christiaan Scott, Kate Webb

**Affiliations:** 1 Department of Paediatrics and Child Health, University of Cape Town, Cape Town, South Africa; 2 Division of Immunology, Institute of Infectious Disease and Molecular Medicine, University of Cape Town, Cape Town, South Africa; 3 Department of Paediatrics and Child Health, Faculty of Medicine and Health Sciences, Stellenbosch University, Tygerberg; 4 Hospital, Cape Town, South Africa; 5 Cape Heart Institute, University of Cape Town, Cape Town, South Africa; 6 The Francis Crick Institute, Crick African Network, London, United Kingdom

## Abstract

**Background:**

Multisystem inflammatory syndrome is a severe manifestation of SARS-CoV-2 in children. The incidence of MIS-C after SARS-CoV-2 infection is poorly understood. There are very few cohorts describing MIS-C in Africa despite MIS-C being more common in Black children worldwide.

**Methods:**

A cohort of children with MIS-C and healthy children was recruited from May 2020 to May 2021 from the two main paediatric hospitals in Cape Town, South Africa. Clinical and demographic data were collected, and serum was tested for SARS-CoV-2 antibodies. The incidence of MIS-C was calculated using an estimation of population exposure from seroprevalence in the healthy group. Summary data, non-parametric comparisons and logistic regression analyses were performed.

**Results:**

Sixty-eight children with MIS-C were recruited with a median age of 7 years and 97 healthy children were recruited with a 30% seroprevalence. The estimated incidence of MIS-C was 22/100 000 SARS-COV-2 infections in children under 14 years old in the city at that time. Black children were over-represented in the MIS-C group (62% *vs* 37%, *p* = 0.002). The most common clinical features in MIS-C were fever (100%), tachycardia (98.5%), rash (85.3%), conjunctivitis (77.9%), abdominal pain (60.3%) and hypotension (60.3%). Median levels of haemoglobin, sodium, CRP, ferritin, cardiac (pro-BNP, trop-T) and coagulation markers (D-dimer and fibrinogen), neutrophil and white cell count were markedly deranged in MIS-C. Cardiac, pulmonary, central nervous and renal organ systems were involved in 71%, 29.4%, 27.9% and 27.9% respectively. Ninety-four point one per cent patient received intravenous immune globulin, 64.7% received methylprednisolone and 61.7% received both. ICU admission was required in 39.7% patient while 38.2% required inotropic support, 38.2% required oxygen therapy, 11.8% required invasive ventilation and 6% required peritoneal dialysis. The median hospital stay duration was 7 days with no deaths.

**Conclusion:**

The lack of reports from Southern Africa does not reflect a lack of cases of MIS-C. The clinical manifestations and outcomes of MIS-C in this region highlight the need for improved surveillance, reporting and data to inform diagnosis and treatment.

**Implications:**

To our knowledge, these are the first data on MIS-C in Africa. This shows that children in Africa are indeed presenting with MIS-C which will increase surveillance around the continent.

